# Risk of post-fracture pneumonia and its association with cardiovascular events and mortality in adults with intellectual disabilities

**DOI:** 10.3389/fpsyt.2023.1208887

**Published:** 2023-11-03

**Authors:** Daniel G. Whitney, Steven R. Erickson, Maryam Berri

**Affiliations:** ^1^Department of Physical Medicine and Rehabilitation, University of Michigan, Ann Arbor, MI, United States; ^2^Institute for Healthcare Policy and Innovation, University of Michigan, Ann Arbor, MI, United States; ^3^Department of Clinical Pharmacy, University of Michigan, Ann Arbor, MI, United States

**Keywords:** intellectual disabilities, fracture, pneumonia, mortality, clinical epidemiology

## Abstract

**Objective:**

Fragility fractures are associated with an increased risk of pneumonia, which is a leading cause of death in adults with intellectual disabilities; however, the timing and complications of post-fracture pneumonia are underinvestigated. The objectives of this study were to determine the 30-day pneumonia rate post-fracture and the association of post-fracture pneumonia with mortality and cardiovascular events among adults with intellectual disabilities.

**Methods:**

This retrospective cohort study was conducted using Medicare and commercial claims from 01 January 2011 to 31 December 2016. Incidence of pneumonia 30 days after a fragility fracture among adults ≥18 years old with intellectual disabilities (Fx cohort) was compared to the incidence among matched adults with intellectual disabilities without fractures (w/oFx cohort) and the general population of patients with an incident fragility fracture (GP+Fx). For the Fx cohort, Cox regression was used to examine the adjusted association of time-varying pneumonia (within 30 days post-fracture) with mortality and incidence of cardiovascular events 0–30, 31–365, and 366–730 days post-fracture.

**Results:**

There was a high–early rate of pneumonia within 30 days post-fracture for young, middle-aged, and elderly adults with intellectual disabilities (*n* = 6,183); this rate was 2.2- to 6.1-fold higher than the rate among the w/oFx (*n* = 12,366) and GP+Fx (*n* = 363,995) cohorts (all *P* < 0.05). For the Fx cohort, post-fracture 30-day incidence of pneumonia was associated with an increased 30-day rate of mortality (adjusted HR [aHR] = 5.19; 95% confidence interval [CI] = 3.68–7.32), heart failure (aHR = 2.96; 95% CI = 1.92–4.56), and cerebrovascular disease (aHF = 1.48; 95% CI = 0.93–2.35; *P* = 0.098), with sustained effects to 1 year for heart failure (aHR = 1.61; 95% CI = 1.19–2.17) and 2 years for mortality (aHR = 1.39; 95% CI = 1.06–1.83), and without evidence of effect modification by age.

**Discussion:**

Adults with intellectual disabilities are vulnerable to post-fracture pneumonia within 30 days, and complications arising from this, across the adult lifespan, and not only during the elderly years.

## 1. Introduction

There is a substantial burden of premature mortality among adults with intellectual disabilities, which is predominately due to cardiorespiratory diseases (1–5). Pneumonia is one of the leading causes of death in adults with intellectual disabilities (4), and fractures may play a role in this.

Bone fragility and fractures are more common and occur earlier in the adult lifespan for individuals with intellectual disabilities as compared to the general population (6–8). Recent work has identified the fact that fragility fractures are associated with an increased rate of mortality and cardiorespiratory diseases among adults aged ≥18 years with developmental and neurological conditions, and especially an increased rate of pneumonia within 30 days post-fracture (9–11). However, the risk of post-fracture pneumonia has not been investigated for adults with intellectual disabilities, which may differ across the adult lifespan.

Post-fracture health decline, especially in relation to pneumonia, are often studied in the general elderly population. Among this group, the rate of pneumonia is highest within 30 days after a fracture or related surgery, which then increases the risk of adverse health and survival outcomes within 30 days and even after this frequently assessed time frame (12–14). The mechanisms linking fractures with a decline in health may stem from the release of pro-inflammatory cytokines into the vasculature (15, 16), which influences the surrounding parenchyma of the cardiorespiratory systems and changes the balance of the coagulation cascade. This series of physiological events can lead to respiratory dysfunction (17), pneumonia (18), and atherosclerosis (19).

A relatively rapid decline in respiratory health post-fracture may have short- and long-term effects on the cardiovascular system. For example, an enhanced risk of cardiovascular morbidity and mortality has been observed after pneumonia, with an increased risk of thrombosis-related vascular disease, such as myocardial infarction and cerebrovascular disease (20). Adults with intellectual disabilities have additional health issues that may further accelerate post-fracture health decline, such as the early development of comorbidities and complex medical profiles (21–24). Therefore, adults with intellectual disabilities may be particularly vulnerable to post-fracture risk of pneumonia and its consequential effects, even prior to reaching the elderly years.

There is a clinical need to better understand the timing and impact of post-fracture pneumonia for individuals with intellectual disabilities across the adult lifespan, and not just in the elderly years. Enhancing this understanding may help physicians to prioritize primary (i.e., fracture) and secondary (i.e., pneumonia) prevention efforts based on the needs of adults with intellectual disabilities. Therefore, the first objective of this study was to characterize the rate of pneumonia within 30 days post-fracture for all adults by age group (to capture rates over the life course) and by fracture site for individuals with intellectual disabilities who sustained a fragility fracture as compared to reference cohorts. The second objective was to examine the association between developing pneumonia within 30 days post-fracture and the rate of mortality and cardiovascular events within up to 2 years of follow-up. Cardiovascular events included congestive heart failure, myocardial infarction, and cerebrovascular disease. The hypothesis was that those who developed pneumonia within 30 days post-fracture would have a higher rate of such outcomes, especially within 30 days and up to 2 years post-fracture, before and after adjusting for possible confounders.

## 2. Methods

### 2.1. Data source

This was a retrospective cohort study that used patient-level claims data from two data sources: (1) a random 20% sample of the Medicare fee-for-service claims database, parts A and B (hospital and medical insurance), but not part C (Medicare Advantage Plan) or D (prescription medication coverage) (hereafter referred to as “Medicare”); and (2) Optum's de-identified Clinformatics^®^ Data Mart Database (hereafter referred to as “Optum”). Medicare is a nationwide federal medical insurance program for all adults ≥65 years old and individuals with end-stage renal disease or certain disabilities (including intellectual disabilities) <65 years old. Optum is a nationwide claims database of individuals privately insured with commercial or Medicare Advantage health plans and provides greater representation of the general adult population <65 years old than Medicare. Information from adults with intellectual disabilities was obtained from the Medicare database. As the 30-day pneumonia rate for the <65 and ≥65-year age groups was examined under Objective 1, information from the cohort without intellectual disabilities, as described below, was obtained from the Optum database to provide a representative background population without intellectual disabilities across the adult lifespan, as previously described (25).

Individuals with Medicare coverage can also have Medicaid coverage. However, Medicare pays first for services that are covered by both Medicare and Medicaid, meaning that this did not impact ascertainment of the variables in this study (25). The medical conditions examined in this study were identified by searching for specific International Classification of Diseases, Ninth or Tenth Revision, Clinical Modification codes (Supplementary Table 1) attached to patient-level claims, which are primarily used for billing reimbursement of healthcare services provided. The University of Michigan Institutional Review Board approved this study as non-regulated because the data were de-identified; patient consent was not required, and this requirement was waived. All methods were carried out in accordance with relevant guidelines and regulations.

### 2.2. Cohort selection

A flowchart showing the processes of inclusion and exclusion to derive the study cohorts is shown in Supplementary Figure 1. For the primary fracture (Fx) cohort, the first fragility fracture event occurring between 01 January 2012 and 31 December 2014 was identified among adults ≥18 years old with intellectual disabilities who had ≥12 months pre-fracture (to obtain baseline information) and ≥1 day post-fracture of continuous enrollment under parts A and B. For this Fx cohort, the index date (time 0) was the fragility fracture date. Patients with intellectual disabilities were defined as those with ≥1 inpatient claim or ≥2 outpatient claims on separate days within 12 months of one another containing a relevant code, which included the most common diagnoses associated with intellectual disabilities (26). Fragility fractures were identified by ≥1 inpatient or outpatient claim for a fracture at an identifiable site without a trauma code (e.g., car accident) 7 days before to 7 days after the index fracture date (9, 27).

There were two comparison cohorts for the first objective: adults with intellectual disabilities who did not sustain a fracture (w/oFx) and adults without intellectual disabilities, drawn from the general population, who sustained an incident fragility fracture (GP+Fx). The index date for the w/oFx cohort was randomly assigned (25). The GP+Fx cohort from the Optum database was developed as part of another study that used the same methodology as described above, but using a different selection sequence and an extended, but overlapping, time period (Supplementary Figure 1) (25).

### 2.3. Pneumonia

Pneumonia was identified on the basis of the presence of ≥1 inpatient or outpatient claim with a relevant code; this method has excellent accuracy, with a positive predictive value of up to 98% (28). Pneumonia in the baseline period was categorized based on the claim date closest to time 0 (i.e., the index date) as 1–14, 15–30, or 31–365 days pre-index. Pneumonia in the follow-up period was identified if the first claim date fell within the period from time 0 to 30 days after time 0.

### 2.4. Outcome events

All-cause mortality was determined using the date of death up to 730 days (2 years) after time 0. Medicare has validated >99% of deaths (29). Cardiovascular events included congestive heart failure, myocardial infarction, and cerebrovascular disease, which were identified on the basis of the presence of ≥1 inpatient or outpatient claim. The single-claim criterion has excellent accuracy, with positive predictive values ranging from 96% to 98% for these outcomes (30). The occurrence of these events in the baseline period was dichotomized (yes/no). For the 2-year follow-up period, the date of the first claim was identified for each cardiovascular event.

### 2.5. Covariates

Information on demographics (i.e., age, sex, race, and U.S. region of residence), dual Medicare–Medicaid eligibility, and the original reason for Medicare entitlement was obtained. Epilepsy was included as it is a common co-occurring neurological condition among individuals with intellectual disabilities and is associated with a high fracture burden and accelerated post-fracture cardiorespiratory health decline (11, 23, 31). Epilepsy was identified in the same manner as intellectual disabilities. A binary variable was constructed to represent evidence of motor dysfunction (yes/no), which included the presence of co-occurring cerebral palsy or spina bifida (identified in the same manner as intellectual disabilities), wheelchair use (wheelchair or accessories), or use of an assistive walking device (e.g., cane) in the baseline period. To account for comorbidity complexity, the Whitney Comorbidity Index (WCI) for the baseline period was calculated. The WCI was developed (32) and validated (33) using administrative claims data for adults with cerebral palsy with or without co-occurring intellectual disabilities and/or epilepsy. The WCI is composed of comorbidities that have greater clinical relevance for adults with intellectual disabilities than other currently available risk indices. Given the study design, this study used a modified version (WCImod), calculated by removing bone fragility, intellectual disabilities, and epilepsy, and summing the presence of 24 clinically relevant comorbidities. All-cause healthcare resource utilization was assessed as the total number of days with a medical encounter during the baseline period (range, 0–365).

### 2.6. Statistical analysis for the first objective

The w/oFx cohort was matched to the Fx cohort to provide meaningful comparisons for the Medicare population of adults with intellectual disabilities. The GP+Fx cohort was not matched, so thatdifferences in cohort-level variables could be identified between adults with and without intellectual disabilities who sustained a fragility fracture. Propensity score-matching of w/oFx to Fx was performed at a 1:2 (case:control) matching ratio, in a random fashion without replacement, and with a caliper of 0.15 standard deviations, using logistic regression based on all covariates listed above, as well as the study entry year, baseline pneumonia, each baseline cardiovascular event, and select WCI comorbidities: chronic pulmonary disease, hypertension, cardiac arrhythmias, renal disease, fluid/electrolyte disorders, diabetes, dysphagia, neurogenic bowel/bladder, and (non-)malignant cancer. The standardized mean difference was used to assess for covariate balance between cohorts, where <0.10 was considered a negligible difference (34).

After matching, baseline descriptive characteristics were summarized for the Fx and w/oFx cohorts. Relevant descriptive characteristics for GP+Fx are reported in the text and Supplementary material due to differences in availability and coding of some variables between Medicare and Optum. The incidence rate (IR per 100 person-months) and the cumulative incidence [using the Fine and Gray approach (35)] of pneumonia within 30 days were estimated for each cohort and then by age group: young (18–40 years), middle-aged (41–64 years), and elderly (≥65 years). The IR ratio (IRR) with 95% confidence intervals (CI) was estimated based on comparison of the Fx cohort to each of the comparison cohorts. Individuals were examined until the day they experienced the pneumonia event or another censoring event; these events were death, loss of continuous health plan enrollment, or the end of the 30-day follow-up period, whichever came first.

A sensitivity analysis was performed after removing individuals who had evidence of pneumonia within 2 weeks pre-index to mitigate bias from possible misclassification of post-index pneumonia as a carryover diagnosis from the baseline period. Another sensitivity analysis was performed in which the Fx and w/oFx cohorts were stratified by the presence of motor dysfunction to determine whether the 30-day pneumonia rate was predominately associated with motor dysfunction.

### 2.7. Statistical analysis for the second objective

The following analyses were performed in the Fx cohort. Cox proportional hazards regression was used to test whether time-varying pneumonia was associated with an increased rate of mortality and incidence of each cardiovascular event (in separate models) during three distinct post-fracture time intervals to assess for short-term and sustained effects: 0–30, 31–365, and 366–730 days post-fracture (14, 25). Pneumonia was examined in the form of time-updated exposure. Adults with evidence of pneumonia within the 2 weeks prior to their fracture date were allocated to the pneumonia-exposed group from time 0. Adults without evidence of pneumonia in the 2 weeks prior to their fracture date but who developed pneumonia within 30 days post-fracture contributed time to the pneumonia-unexposed group from time 0 to the day before their pneumonia diagnosis date, and then contributed time to the pneumonia-exposed group thereafter until the end of the 30-day time period or censor date if <30 days, whichever came first (25). Pneumonia >30 days post-fracture was not part of this analysis. The effect size is reported in the form of a hazard ratio (HR).

Models were developed before and after adjusting for the following possible confounders: age, sex, co-occurring epilepsy, motor dysfunction, fracture site, WCImod, and baseline pneumonia (15–365 days pre-fracture). Confounder selection was guided by recommendations using the “disjunctive cause criterion” (36). Adults were examined up to the day of the outcome event, death when modeling cardiovascular events, loss of continuous health plan enrollment, or end of the follow-up period, whichever came first. Adults who had an occurrence of a specific cardiovascular event in the baseline period (e.g., cerebrovascular disease) were excluded from the specific analysis examining that same cardiovascular event (e.g., post-fracture cerebrovascular disease) in order to restrict the analysis to incident events. Individuals were excluded from the 31–365 and 366–730-day time interval analyses if they experienced the event or were lost to follow-up in the preceding time interval(s). The proportional hazards assumption was tested based on weighted Schoenfeld residuals. Effect modification by age, sex, fracture site, and motor dysfunction was tested in the adjusted model.

Additional models were examined as sensitivity analyses to assess the robustness of the primary models; these models further adjusted for dual Medicare–Medicaid and baseline healthcare resource utilization. These analyses were interpreted according to the change in the effect estimate of time-varying pneumonia from the primary models to assess for possible confounding.

Analyses were performed using SAS version 9.4, and *P* < 0.05 (two-tailed) was considered statistically significant.

## 3. Results

There were 74,025 adults with intellectual disabilities who were eligible for entry into the analysis, of whom 8.4% had a fragility fracture (*n* = 6,183). Of the remaining 67,842 adults with intellectual disabilities who did not sustain a fracture, 12,366 were successfully matched to the Fx cohort (1:2 matching ratio) without losing individuals from the Fx cohort and with standardized mean differences of <0.02 for all covariates.

The baseline characteristics of the matched Fx and w/oFx cohorts are presented in Table 1. The prevalence of each WCI comorbidity not shown in Table 1 is presented in Supplementary Table 2. Compared to the GP+Fx cohort (*n* = 363,995, baseline characteristics presented in Supplementary Table 3), the Fx cohort was on average 7.3 years younger (mean age, 58.6 vs. 65.9 years), had a lower proportion of women (54.3% vs. 65.5%), had a higher WCImod (median [interquartile range], 4 [2–7] vs. 3 [1–5]), and had a different fracture distribution by site; e.g., for GP+Fx, the distribution included 27.1% at the vertebral column, 17.7% at the hip, 2.6% at the non-proximal femur, and 23.4% at the tibia/fibula.

**Table 1:** Baseline characteristics of adults with intellectual disabilities who had sustained a fragility fracture (Fx) and propensity score-matched adults with intellectual disabilities without a fracture (w/oFx) (1:2 matching ratio).

	**Fx**	**w/oFx**
	**(*n* = 6,183)**	**(*n* = 12,366)**
Age, mean (SD)	58.6 (13.5)	58.7 (14.0)
18–40 years, % (*n*)	9.8 (603)	10.6 (1,315)
41–64 years, % (*n*)	59.1 (3,654)	56.6 (6,995)
≥65 years, % (*n*)	31.2 (1,926)	32.8 (4,056)
**Sex, % (** ***n*** **)**		
Women	54.3 (3,355)	54.0 (6,679)
Men	45.7 (2,828)	46.0 (5,687)
**Race, % (** ***n*** **)**		
Asian	0.6 (36)	1.0 (128)
Black	8.5 (527)	10.7 (1,324)
Hispanic	1.8 (110)	3.2 (390)
North American Native	0.7 (42)	0.9 (114)
White	87.3 (5,400)	82.3 (10,180)
Other	1.1 (68)	1.9 (230)
**U.S. region of residence, % (** ***n*** **)**		
Northeast	24.7 (1,524)	25.0 (3,091)
Midwest	28.9 (1,786)	29.1 (3,602)
South	34.1 (2,111)	33.3 (4,119)
West	12.3 (762)	12.6 (1,554)
**Original reason for Medicare entitlement, % (** ***n*** **)**		
Old age and survivor's insurance	7.0 (433)	7.1 (881)
Disability insurance benefits (DIB)	92.4 (5,713)	92.1 (11,389)
End-stage renal disease (ESRD)	0.1 (6)	0.2 (24)
Both DIB and ESRD	0.5 (31)	0.6 (72)
**Dual eligibility with Medicaid, % (** ***n*** **)**		
Full	88.8 (5,493)	88.8 (10,982)
Partial	2.9 (176)	2.9 (353)
None	8.3 (514)	8.3 (1, 031)
**Severity of intellectual disability, % (** ***n*** **)**		
Mild	20.2 (1,248)	20.7 (2,559)
Moderate	8.9 (550)	8.3 (1, 029)
Severe or profound	11.6 (718)	12.5 (1,550)
Other/unspecified	59.3 (3,667)	58.5 (7,228)
Co-occurring epilepsy, % (*n*)	34.8 (2,154)	35.2 (4,355)
Motor dysfunction, % (*n*)	20.4 (1,259)	20.3 (2,514)
Cerebral palsy, % (*n*)	12.0 (741)	12.2 (1,512)
Spina bifida, % (*n*)	0.4 (25)	0.2 (29)
Wheelchair, % (*n*)	9.6 (595)	9.6 (1,183)
Assistive walking device, % (*n*)	2.4 (151)	2.0 (242)
**Modified Whitney comorbidity index**		
Median (IQR)	4 (2–7)	4 (2–7)
**All-cause healthcare resource utilization**		
Median (IQR)	32 (14–91)	29 (12–89)
**Fracture site, % (** ***n*** **)**		
Vertebral column	19.6 (1,211)	-
Hip	19.1 (1,179)	-
Non-proximal femur	4.5 (276)	-
Tibia/fibula	27.9 (1,725)	-
Humerus	10.4 (644)	-
Radius/ulna	11.5 (710)	-
Multiple sites	7.1 (438)	-
**Outcome occurrence during baseline period, % (** ***n*** **)**		
**Pneumonia**		
No	83.7 (5,173)	82.7 (10,230)
1–12 months pre-index	11.6 (714)	13.2 (1,626)
2–4 weeks pre-index	1.9 (117)	1.6 (201)
1–14 days pre-index	2.9 (179)	2.5 (309)
Congestive heart failure	14.3 (885)	14.0 (1,731)
Myocardial infarction	1.8 (113)	1.7 (204)
Cerebrovascular disease	15.2 (937)	15.0 (1,852)

SD, standard deviation; IQR, interquartile range.

### 3.1. Objective #1: 30-day rate of pneumonia

**Figure 1:**
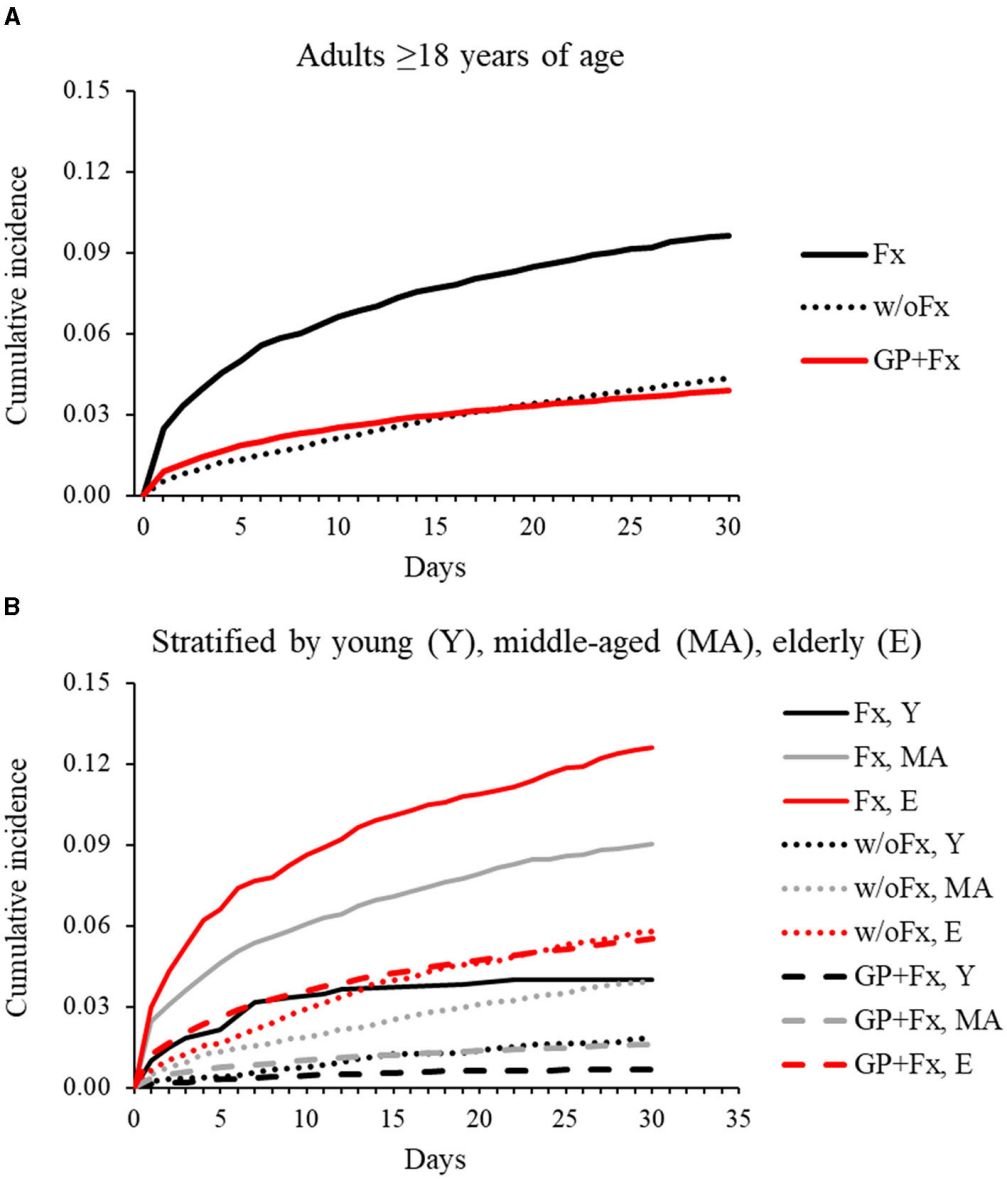
Cumulative incidence of pneumonia over 30 days for adults with intellectual disabilities who had sustained a fragility fracture (Fx; *n* = 6,183) and comparison cohorts: matched adults with intellectual disabilities who had not sustained a fracture (w/oFx; *n* = 12,366) and the general population of adults without intellectual disabilities who had sustained a fragility fracture (GP+Fx; *n* = 363,995). **(A)** Represents the full cohorts ≥18 years of age. **(B)** Represents the cohorts after stratification into young (18–40 years), middle-aged (41–64 years), and elderly (≥65 years) groups.

The elderly group within the GP+Fx cohort had a high and early rate of pneumonia within 30 days (IR = 5.9 per 100 person-months; 95% CI = 5.8–6.0), with the pattern and magnitude being considerably different from those of the young (IR = 0.7; 95% CI = 0.6–0.8) and middle-aged (IR = 1.6; 95% CI = 1.5–1.7) groups (Figure 1, Table 2). Analysis of 30-day pneumonia rates for the Fx cohort also showed a high–early pattern, but this was evident for all age groups and not just the elderly. Furthermore, the 30-day pneumonia rate for the young (IR = 4.2; 95% CI = 2.5–5.8), middle-aged (IR = 9.8; 95% CI = 8.8–10.9), and elderly (IR = 14.1; 95% CI = 12.3–15.9) groups was higher for the Fx cohort compared to the w/oFx and GP+Fx cohorts within the same age strata. For example, IRRs comparing the Fx cohort to the comparison cohorts ranged from 2.23 to 6.11 (all *P* < 0.05) (Table 2). The cumulative incidence of pneumonia within 30 days by fracture site is presented in Supplementary Figure 2. The 30-day pneumonia rate was higher for non-elderly and elderly people in the Fx cohort as compared to elderly people in the GP+Fx cohort for each fracture site, especially for vertebral column, hip, and non-proximal femur fractures.

**Table 2:** Thirty-day incidence rate (IR) and IR ratio (IRR) of pneumonia for adults with intellectual disabilities who had sustained a fragility fracture (Fx) and comparison cohorts: propensity score-matched adults with intellectual disabilities who had not sustained a fracture (w/oFx), and the general population of adults without intellectual disabilities who had sustained a fragility fracture (GP+Fx).

	**Sample size (*n*)**	**Pneumonia events % (*n*)**	**Person-months**	**IR (95% CI)**	**IRR^a^ (95% CI)**
**All ages**, ≥**18 years**				
Fx	6,183	9.6 (593)	5,623	10.5 (9.7, 11.4)	-
w/oFx, post-match	12,366	4.3 (531)	11,790	4.5 (4.1, 4.9)	2.34 (2.08, 2.63)
GP+Fx	363,995	3.8 (13,955)	343,320	4.1 (4.0, 4.1)	2.59 (2.39, 2.82)
**Young, 18–40 years**				
Fx	603	4.0 (24)	575	4.2 (2.5, 5.8)	-
w/oFx, post-match	1,315	1.8 (24)	1,282	1.9 (1.1, 2.6)	2.23 (1.27, 3.92)
GP+Fx	42,639	0.7 (281)	41,165	0.7 (0.6, 0.8)	6.11 (4.03, 9.27)
**Middle-aged, 41–64 years**				
Fx	3,654	9.0 (328)	3,341	9.8 (8.8, 10.9)	-
w/oFx, post-match	6,995	3.9 (274)	6,695	4.1 (3.6, 4.6)	2.40 (2.04, 2.82)
GP+Fx	99,150	1.6 (1,560)	95,157	1.6 (1.5, 1.7)	5.99 (5.32, 6.75)
**Elderly**, ≥**65 years**				
Fx	1,926	12.5 (241)	1,707	14.1 (12.3, 15.9)	-
w/oFx, post-match	4,056	5.7 (233)	3,814	6.1 (5.3, 6.9)	2.31 (1.93, 2.77)
GP+Fx	222,206	5.5 (12,114)	206,998	5.9 (5.8, 6.0)	2.41 (2.12, 2.74)

CI, confidence interval.

a The estimate compares Fx to the corresponding comparison group; e.g., the IRR of 2.59 represents a comparison of the Fx rate to the w/oID+Fx rate.

A sensitivity analysis excluding individuals with evidence of pneumonia within 2 weeks of their index date resulted in slightly lower IRs for all cohorts but similar conclusions in all comparisons. For example, the IR was 8.9 per 100 person-months (95% CI = 8.1–9.6) for the Fx cohort; this remained elevated compared to the w/oFx cohort (IRR = 2.83; 95% CI = 2.47–3.25) (data not shown).

For the sensitivity analysis in which the Fx and w/oFx cohorts were stratified according to presence vs. absence of motor dysfunction, the IR for pneumonia within 30 days is presented in Supplementary Table 4, and the cumulative incidence plot is shown in Supplementary Figure 3. The IR for pneumonia within 30 days was higher for those with vs. without motor dysfunction for both cohorts (Fx, w/oFx) for the young, middle-aged, and elderly age groups. The IR was similar for the Fx cohort without motor dysfunction and the w/oFx cohort with motor dysfunction in the case of the young and middle-aged groups, but higher in the Fx cohort in the case of the elderly agegroup.

### 3.2. Objective #2: consequences of post-fracture pneumonia

Over the 2-year post-fracture follow-up period for the Fx cohort, 17.7% of patients died (IR for the entire 2-year period = 19.9; 95% CI = 18.7–21.1), 15.9% had incident congestive heart failure (IR = 19.2; 95% CI = 17.9–20.5), 3.6% had incident myocardial infarction (IR = 4.1; 95% CI = 3.5–4.6), and 20.1% had incident cerebrovascular disease (IR = 25.3; 95% CI = 23.8–26.8). The proportion and IR of mortality and cardiovascular outcomes at the distinct time intervals are presented in Supplementary Table 5. To enhance model parsimony, patients were grouped into three categories by fracture site based on the association with 30-day and 31–365-day mortality (data not shown): (1) non-proximal femur; (2) vertebral column, hip, or multiple simultaneous sites; and (3) tibia, fibula, humerus, radius, or ulna.

Developing pneumonia within 30 days post-fracture was associated with an increased rate of mortality, with stronger associations in the first 30 days post-fracture (aHR = 5.19; 95% CI = 3.68–7.32) compared to 31–365 days (aHR = 2.10; 95% CI = 1.70–2.59) and 366–730 days (aHR = 1.39; 95% CI = 1.06–1.83) post-fracture (Table 3). The proportional hazards assumption was violated for time-varying pneumonia when modeling 31–365-day mortality (*P* < 0.001). The fitted penalized B-spline curve was assessed using the adjusted model, which measures the time dependency of the relationship; the results suggested that time-varying pneumonia had a stronger association with mortality rate earlier during the time interval (Supplementary Figure 4).

**Table 3:** Association between time-varying pneumonia and outcomes at distinct time intervals post-fracture for adults with intellectual disabilities.

	**Model sample size**	**Unadjusted**	**Adjusted**
	**N**	**HR (95% CI)**	**HR (95% CI)**
**30-day outcomes**			
Mortality	6,183	7.90 (5.70, 10.94)	5.19 (3.68, 7.32)
Congestive heart failure	5,298	3.82 (2.52, 5.80)	2.96 (1.92, 4.56)
Myocardial infarction	6,070	2.80 (1.24, 6.31)	^a^
Cerebrovascular disease	5,246	1.93 (1.23, 3.01)	1.48 (0.93, 2.35)^c^
**31–365-day outcomes**			
Mortality	6,030	3.04 (2.48, 3.71)^b^	2.10 (1.70, 2.59)^b^^,^^d^
Congestive heart failure	5,033	2.19 (1.64, 2.92)	1.61 (1.19, 2.17)^d^
Myocardial infarction	5,883	1.88 (1.04, 3.39)	1.19 (0.65, 2.20)
Cerebrovascular disease	4,945	1.56 (1.20, 2.04)	1.05 (0.80, 1.39)
**366–730-day outcomes**			
Mortality	5,489	2.10 (1.61, 2.73)	1.39 (1.06, 1.83)^c^
Congestive heart failure	4,357	1.42 (0.93, 2.18)	0.94 (0.61, 1.46)
Myocardial infarction	5,321	1.15 (0.56, 2.38)	0.63 (0.30, 1.33)
Cerebrovascular disease	4,107	1.38 (0.96, 2.00)	0.98 (0.67, 1.43)

HR, hazard ratio; CI, confidence interval. The adjusted model included the following covariates: age, sex, epilepsy, motor dysfunction, fracture site, Whitney Comorbidity Index, and baseline occurrence of pneumonia from 2 weeks to 1 year pre-index.

a Very few events for analysis.

b Violated proportional hazards assumption.

c A statistically significant interaction between time-varying pneumonia and sex.

d A statistically significant interaction between time-varying pneumonia and fracture site.

Developing pneumonia within 30 days post-fracture was associated with an increased rate of congestive heart failure at the 30-day and 31–365-day time intervals, and with 30-day incidence of cerebrovascular disease, but the latter was statistically insignificant (aHR = 1.48; 95% CI = 0.93–2.35; *P* = 0.098). There were too few incident myocardial infarction events for the adjusted 30-day analysis.

There was evidence of effect modification by sex for 30-day incidence of cerebrovascular disease and 366–730-day mortality; by fracture site for 31–365-day congestive heart failure and mortality; and by motor dysfunction for 31–365-day mortality (*P* for interaction, 0.026–0.041). The adjusted relative rate (i.e., HR) for relevant outcomes by time-varying pneumonia within 30 days post-fracture was higher for women than men; for fractures at the tibia, fibula, humerus, radius, or ulna compared to other grouped fracture sites; and for those without vs. with motor dysfunction (Supplementary Table 6).

The conclusions drawn in the primary analysis were unchanged in the sensitivity analyses that involved further adjustment for dual Medicare–Medicaid and baseline healthcare resource utilization (data not shown). For example, the association of time-varying pneumonia with 30-day incidence of cerebrovascular disease (HR = 1.51; 95% CI = 0.95–2.41), 31–365-day congestive heart failure (HR = 1.64; 95% CI = 1.22–2.20), and 366–730-day mortality (HR = 1.38; 95% CI = 1.05–1.82) was similar to the association observed in the primary analysis in each case.

## 4. Discussion

This study indicates that adults with intellectual disabilities, with and without motor dysfunction, have an increased risk of pneumonia within 30 days post-fracture across the adult lifespan and not just in the elderly years. In addition, developing pneumonia within 30 days post-fracture was associated with a substantially increased rate of mortality and cardiovascular events within 30 days, and with increased rates of congestive heart failure over the course of up to 1 year of follow-up and mortality over the course of up to 2 years of follow-up.

The 30-day post-fracture pneumonia rate observed in the GP+Fx cohort was consistent with expectations, in that the elderly group, but not the young or middle-aged groups, had a high and early rate (12–14, 37). For adults with intellectual disabilities, there was a high–early 30-day rate of pneumonia post-fracture for the elderly group, but also for the young and middle-aged groups. While evidence of motor dysfunction was associated with a higher 30-day pneumonia rate, adults with intellectual disabilities without motor dysfunction still had substantially elevated 30-day pneumonia rates post-fracture across the adult lifespan. This suggests that the 30-day risk of pneumonia post-fracture is not only an issue for the elderly or those with motor dysfunction among adults with intellectual disabilities.

The inclusion of two comparison cohorts broadens the clinical implications of the study findings. For clinicians who treat adults who have sustained a fragility fracture, it is useful to recognize that patients with vs. without intellectual disabilities will be more likely to develop pneumonia within 30 days, regardless of age. While the absolute rate was higher for older ages, the relative rate for those with vs. without intellectual disabilities who had sustained a fragility fracture was ~6-fold higher for young and middle-aged adults and 2.4-fold higher for elderly adults. For clinicians who treat adults with intellectual disabilities regardless of clinical specialty, the comparison to the w/oFx cohort provides important insights. This study used a propensity score-matched design to account for cohort differences, possible confounding by demographics, and multiple indicatoedrs of medical complexity. The results suggest that sustaining a fragility fracture is associated with a 2.2- to 2.4-fold high rate of pneumonia within 30 days among adult patients with intellectual disabilities.

This study also found that peri/post-fracture pneumonia risk within 30 days was associated with higher mortality and cardiovascular event rates, especially in the short term (30 days), with sustained effects on congestive heart failure up to 1 year post-fracture and mortality up to 2 years post-fracture. There was no evidence of effect modification by age, suggesting that the consequential effect of peri/post-fracture pneumonia is a problem for individuals with intellectual disabilities across the adult lifespan and not only among the elderly. However, there was evidence of effect modification by other variables; these are important to highlight in order to enhance clinical interpretations. First, pneumonia within 30 days post-fracture was associated with an increased rate of cerebrovascular disease, but only for women and at 30 days post-fracture. This highlights a unique sub-cohort at risk for short-term cerebrovascular disease following a fragility fracture. Second, pneumonia within 30 days post-fracture was associated with an increased 366–730-day mortality rate in women but not in men. These findings suggest that post-fracture pneumonia is strongly associated with short-term 30-day mortality among men and women with intellectual disabilities, but the sustained effect diminishes more for men than women after 1 year post-fracture.

The limitations that may directly impact the conclusions of this study must be discussed. It was not possible to study associations according to the severity of intellectual disabilities because almost half of the members of the cohort had an “unspecified” type of intellectual disability. However, this lack of information is not expected to alter the broad conclusions drawn because the models were adjusted for covariates that can act as proxy variables for severity, such as epilepsy, motor dysfunction, and modWCI, which captured relevant (multi-)morbidity profiles. The pneumonia variable included all types of pneumonia. Adults with intellectual disabilities may have differential risk of particular pneumonia subtypes and subsequent cardiorespiratory complications. Given the observational design, the findings relating to the second objective are subject to bias from unmeasured and residual confounding. There are relevant confounders that are not available in claims data, such as smoking, anthropometrics, and lifestyle factors (e.g., physical activity, alcohol consumption, and stress), or that the study team did not have access to, such as Medicare part D for prescription medications. While this study adjusted for several indicators of medical complexity, the variables were dichotomized, which does not capture the differing severities and effects of conditions (e.g., epilepsy and motor dysfunction) or whether these indicators of medical complexity were clinically managed or uncontrolled (e.g., diabetes). Finally, the generalizability of the study findings to the broader population of adults with intellectual disabilities is unknown. The findings should therefore be interpreted within the context of this Medicare cohort.

The findings of this study can inform clinical priorities for primary (e.g., fragility fracture) and secondary (e.g., post-fracture pneumonia) post-fracture prevention efforts among adults with intellectual disabilities. Cardiorespiratory diseases, including pneumonia, are among the leading causes of death in adults with intellectual disabilities (1–5). Sustaining a fragility fracture increases the risk for pneumonia within 30 days, which in turn is strongly associated with an increased risk of mortality up to 2 years and cardiovascular events up to 1 year post-fracture, and especially within 30 days post-fracture.

## Data availability statement

The data analyzed in this study is subject to the following licenses/restrictions: The data that support the findings of this study are available from Centers for Medicare & Medicaid Services and Optum's de-identified Clinformatics^®^ Data Mart Database but restrictions apply to the availability of these data, which were used under license for the current study, and so are not publicly available. Requests to access these datasets should be directed to https://www.cms.gov/.

## Author contributions

DW conceptualized the study, analyzed the data, and wrote the first draft of the manuscript. DW, SE, and MB designed the study, approved the final version of this manuscript, and agree to be accountable for the content of the work. SE and MB edited the manuscript. All authors contributed to the article and approved the submitted version.
