# Supplementary material for: Risk of post-fracture pneumonia and its association with cardiovascular events and mortality in adults with intellectual disabilities
Source: Front Psychiatry. 2023 Nov 3;14:1208887. doi: 10.3389/fpsyt.2023.1208887 (PMC10654739; doi:10.3389/fpsyt.2023.1208887)

Supplementary Material

Post-fracture pneumonia risk and association with cardiovascular events and mortality for adults with intellectual disabilities

Daniel G. Whitney*, Steven R. Erickson, Maryam Berri

*** Correspondence:** Corresponding Author: dgwhit@umich.edu

Supplementary Table 1. International Classification of Diseases, Ninth (ICD-9) and Tenth (ICD-10) Revision, Clinical Modification and Healthcare Common Procedure Coding System (HCPCS) codes to identify variables for this study.

	ICD-9 codes	ICD-10 codes	HCPCS codes
**Primary cohort**			
Intellectual disabilities	270.1x, 317.x-319.x, 758.0, 758.1, 759.81, 760.71 759.83		
**Motor dysfunction**			
Cerebral palsy, spina bifida, wheelchair and accessories, assistive walking device	333.71, 343.0-343.4, 343.8, 343.9, 741.x, 756.17, V46.3		E0100, E0105, E0130, E0135, E0140-E0144, E0147-E0149, E0154-E0159, E095.x-E099.x, E10.x-E12.x, E22.x, E23.x, E26.x, K000.x-K01.x, K0813-K0899
**Whitney Comorbidity Index comorbidities**			
Hypertension (un)complicated	401., 401.9, 402.10, 402.90, 404.10, 404.90, 405.11, 405.19, 405.91, 405.99		
Epilepsy	345.x		
Chronic pulmonary disease	416.8, 416.9, 490.x-505.x, 506.4, 508.1, 508.8, 518.83		
Depression	300.4, 301.12, 309.0, 309.1, 311		
Blood loss and deficiency anemias	280.x-281.9, 285.9		
Gastrointestinal issues	531.70, 531.90, 532.70, 532.90, 533.70, 533.90, 534.70, 534.90, 556.x, 564.0x, 564.1, V12.71		
Osteoarthritis and allied disorders	715.x		
Intellectual disabilities	317.x-319.x		
Fluid and electrolyte disorders	276.0-276.9		
Cardiac arrhythmias	426.10, 426.11, 426.13, 426.2-426.53, 426.6-426.89, 427.0, 427.2, 427.31, 427.60, 427.9, 785.0, V45.0, V53.3		
Hypothyroidism	243-244.2, 244.8, 244.9		
Dysphagia	787.2x		
Diabetes without chronic complication	250.0-250.3, 250.8, 250.9		
Other neurological disorders	331.9, 332.0, 333.4, 333.5, 334.0-335.9,		

	340, 341.1-341.9, 348.1, 348.3, 780.3, 784.3		
Renal disease	403.01, 403.11, 403.91, 404.02, 404.03, 404.12, 404.13, 404.92, 404.93, 582.x, 583.0-583.7, 585.x, 586.x, 588.0, V42.0, V45.1, V56.x		
Any malignancy, including lymphoma and leukemia, except malignant neoplasm of skin	140.x-172.x, 174.x-195.8, 200.x-208.x, 238.6, V10.00-V10.9		
Diabetes with chronic complication	250.4-250.7		
Neurogenic bowel or bladder	564.81, 596.54		
Mild to severe liver disease	070.22, 070.23, 070.32, 070.33, 070.44, 070.54, 070.6, 070.9, 456.0-456.2, 570.x, 571.x, 572.2-572.8, 573.3, 573.4, 573.8, 573.9, V42.7		
Dementia	290.x, 294.1, 331.0, 331.2		
Myocardial infarction for baseline	410.x, 412.x		
Rheumatoid arthritis and other inflammatory polyarthropathies	714.x		
Metastatic cancer	196.x-199.1		
**Outcomes**			
Pneumonia	480.x-486.x	J13, J14, J15.x, J16.x, J17, J18.x	
Congestive heart failure	398.91, 402.11, 402.91, 404.11, 404.13, 404.91, 404.93, 428.0-428.9	I09.9, I11.0, I13.0, I13.2, I25.5, I42.0, I42.5-I42.9, I43.x, I50.x, P29.0	
Myocardial infarction for follow-up	410.x	I21.x, I22.x	
Cerebrovascular disease	362.34, 430.x-438.x	G45.x, G46.x, H34.0, I60.x-I69.x	

Supplementary Table 2. Prevalence of Whitney Comorbidity Index comorbidities for adults with intellectual disabilities that sustained a fragility fracture (Fx) and propensity score matched adults with intellectual disabilities without a fracture (w/oFx) (1:2 matching ratio).

	Fx (n=6,183)	w/oFx (n=12,366)
	%	%
Hypertension	52.7	52.3
Other neurological disorders, excluding epilepsy	39.8	38.2
Blood loss anemia	35.4	34.9
Hypothyroidism	33.7	34.3
Diabetes without complications	29.8	30.2
Fluid and electrolyte disorders	27.8	28.0
Chronic pulmonary disease	26.5	26.1
Osteoarthritis	26.5	21.4
Cardiac arrhythmias	25.5	25.1
Depression	25.0	25.0
Gastrointestinal issues	25.0	25.3
Dysphagia	16.5	17.0
Dementia/Alzheimer's disease	11.8	13.0
Renal disease	11.4	11.4
Diabetes with complications	10.7	10.6
Any malignancy, including lymphoma and leukemia, except malignant neoplasm of skin	10.1	10.1
Liver disease	6.8	6.8
Neurogenic bowel or bladder	3.2	3.0
Rheumatoid arthritis	2.4	2.1
Metastatic cancer	1.4	1.4

Supplementary Table 3. Baseline characteristics of adults without cerebral palsy that sustained a fragility fracture (n=363,995).

	% (n)
Age, mean (SD)	65.9 (18.3)
≥65 years	61.1 (222,206)
Sex	
Female	65.5 (238,288)
Male	34.5 (125,707
Race	
Asian	2.5 (9,011)
Black	6.3 (23,013)
Hispanic	8.2 (29,913)
White	68.5 (249,473)
Other/missing	14.5 (52,585)
U.S. region of residence	
Northeast	28.2 (102,792)
Midwest	24.7 (89,717)
South	35.6 (129,512)
West	11.5 (41,974)
Whitney Comorbidity Index	
Median (IQR)	3 (1-5)
Fracture site	
Hip	17.7 (64,465)
Vertebral column	27.1 (98,603)
Non-proximal femur	2.6 (9,489)
Tibia/fibula	23.4 (85,019)
Humerus	10.4 (37,885)
Forearm	18.8 (68,534)

SD, standard deviation; IQR, interquartile range.

Supplementary Table 4. 30-day incidence rate (IR) of pneumonia for adults with intellectual disabilities that sustained a fragility fracture (Fx) stratified by the presence of motor dysfunction, and comparison cohorts: propensity score matched adults with intellectual disabilities that did not sustain a fracture (w/oFx) and the general population of adults without intellectual disabilities that sustained a fragility fracture (GP+Fx).

	Pneumonia events % (n)	IR per 100 person months (95% CI)
**Young, 18-40 years**		
Fx	4.0 (24)	4.2 (2.5, 5.8)
With motor dysfunction	8.5 (10)	9.3 (3.5, 15.1)
Without motor dysfunction	2.9 (14)	3.0 (1.4, 4.6)
w/oFx, post-match	1.8 (24)	1.9 (1.1, 2.6)
With motor dysfunction	2.9 (7)	3.0 (0.8, 5.2)
Without motor dysfunction	1.6 (17)	1.6 (0.9, 2.4)
GP+Fx	0.7 (281)	0.7 (0.6, 0.8)
**Middle-aged, 41-64 years**		
Fx	9.0 (328)	9.8 (8.8, 10.9)
With motor dysfunction	14.1 (109)	15.8 (12.9, 18.8)
Without motor dysfunction	7.6 (219)	8.3 (7.2, 9.3)
w/oFx, post-match	3.9 (274)	4.1 (3.6, 4.6)
With motor dysfunction	7.4 (103)	8.0 (6.4, 9.5)
Without motor dysfunction	3.1 (171)	3.2 (2.7, 3.6)
GP+Fx	1.6 (1,560)	1.6 (1.5, 1.7)
**Elderly, ≥65 years**		
Fx	12.5 (241)	14.1 (12.3, 15.9)
With motor dysfunction	15.9 (58)	18.4 (13.7, 23.1)
Without motor dysfunction	11.7 (183)	13.2 (11.2, 15.1)
w/oFx, post-match	5.7 (233)	6.1 (5.3, 6.9)
With motor dysfunction	9.0 (79)	9.9 (7.7, 12.1)
Without motor dysfunction	4.9 (154)	5.1 (4.3, 5.9)
GP+Fx	5.5 (12,114)	5.9 (5.8, 6.0)

CI, confidence interval.

Supplementary Table 5. Incidence rate (IR) of mortality and cardiovascular outcomes at distinct time intervals post-fracture for adults with intellectual disabilities.

**30-day outcomes**	Sample size (n)	Incident events % (n)	Person-months	IR (95% CI)
Mortality	6,183	2.5 (153)	6,020	2.5 (2.1, 2.9)
Congestive heart failure	5,298	3.1 (165)	5,058	3.3 (2.8, 3.8)
Myocardial infarction	6,070	0.8 (46)	5,880	0.8 (0.6, 1.0)
Cerebrovascular disease	5,246	3.8 (200)	4,996	4.0 (3.4, 4.6)
**31-365 day outcomes**	Sample size (n)	Incident events % (n)	Person-years	IR (95% CI)
Mortality	6,030	8.9 (538)	5,750	9.4 (8.6, 10.1)
Congestive heart failure	5,033	7.9 (399)	4,688	8.5 (7.7, 9.3)
Myocardial infarction	5,883	1.4 (85)	5,593	1.5 (1.2, 1.8)
Cerebrovascular disease	4,945	10.6 (524)	4,509	11.6 (10.6, 12.6)
**366-730 day outcomes**	Sample size (n)	Incident events % (n)	Person-years	IR (95% CI)
Mortality	5,489	7.4 (405)	10,735	3.8 (3.4, 4.1)
Congestive heart failure	4,357	6.4 (278)	8,438	3.3 (2.9, 3.7)
Myocardial infarction	5,321	1.6 (87)	10,386	0.8 (0.7, 1.0)
Cerebrovascular disease	4,107	8.0 (328)	7,899	4.2 (3.7, 4.6)

CI, confidence interval.

Supplementary Table 6. Association between time-varying pneumonia within 30-days post-fracture (reference: no pneumonia within 30-days) and outcomes with evidence of effect modification by sex or fracture site for adults with intellectual disabilities.

	30-day cerebrovascular disease	31-365 day congestive heart failure	31-365 day mortality	366-730 day mortality
	HR (95% CI)	HR (95% CI)	HR (95% CI)	HR (95% CI)
**Sex**				
Women	2.31 (1.31, 4.08)	-	-	1.80 (1.27, 2.57)
Men	0.83 (0.38, 1.82)	-	-	1.03 (0.67, 1.56)
**Fracture site**				
Femur, non-proximal	-	1.95 (0.71, 5.33)	1.06 (0.44, 2.57)	-
Vertebrae, hip, multiple sites	-	1.16 (0.77, 1.75)	1.93 (1.50, 2.48)	-
Tibia, fibula, humerus, radius, ulna	-	2.66 (1.69, 4.18)	3.09 (2.12, 4.51)	-
**Motor dysfunction**				
No	-	-	2.37 (1.88, 3.00)	-
Yes	-	-	1.43 (0.93, 2.20)	-

HR, hazard ratio; CI, confidence interval. The models are adjusted for age, sex (except when stratified by sex), epilepsy, motor dysfunction, fracture site (except when stratified by fracture site), Whitney Comorbidity Index, and baseline pneumonia from 2-weeks to 1-year pre-index. The effect estimate (i.e., HR) represents the association between those with vs. without pneumonia exposure within 30-days post-fracture for that patient factor.

Supplementary Figure 1. Flow chart of inclusion/exclusion criteria from the Medicare database to obtain adults with intellectual disabilities that sustained an incident fragility fracture and adults with intellectual disabilities that did not sustain a fracture, and from the Optum database to obtain the general population of adults without intellectual disabilities that sustained an incident fragility fracture.

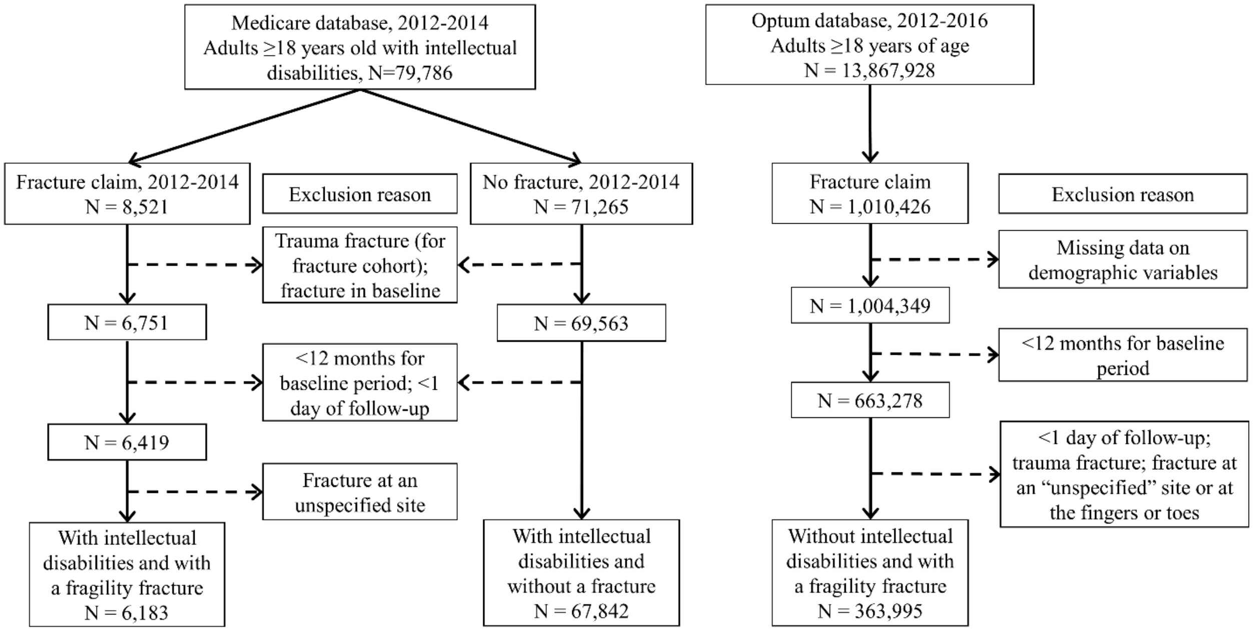

Supplementary Figure 2. Cumulative incidence of pneumonia over 30 days post-fracture by fragility fracture site for non-elderly (<65 years) and elderly (≥65 years) adults with intellectual disabilities (Fx; n=6,183) and the general elderly population without intellectual disabilities (GP+Fx).

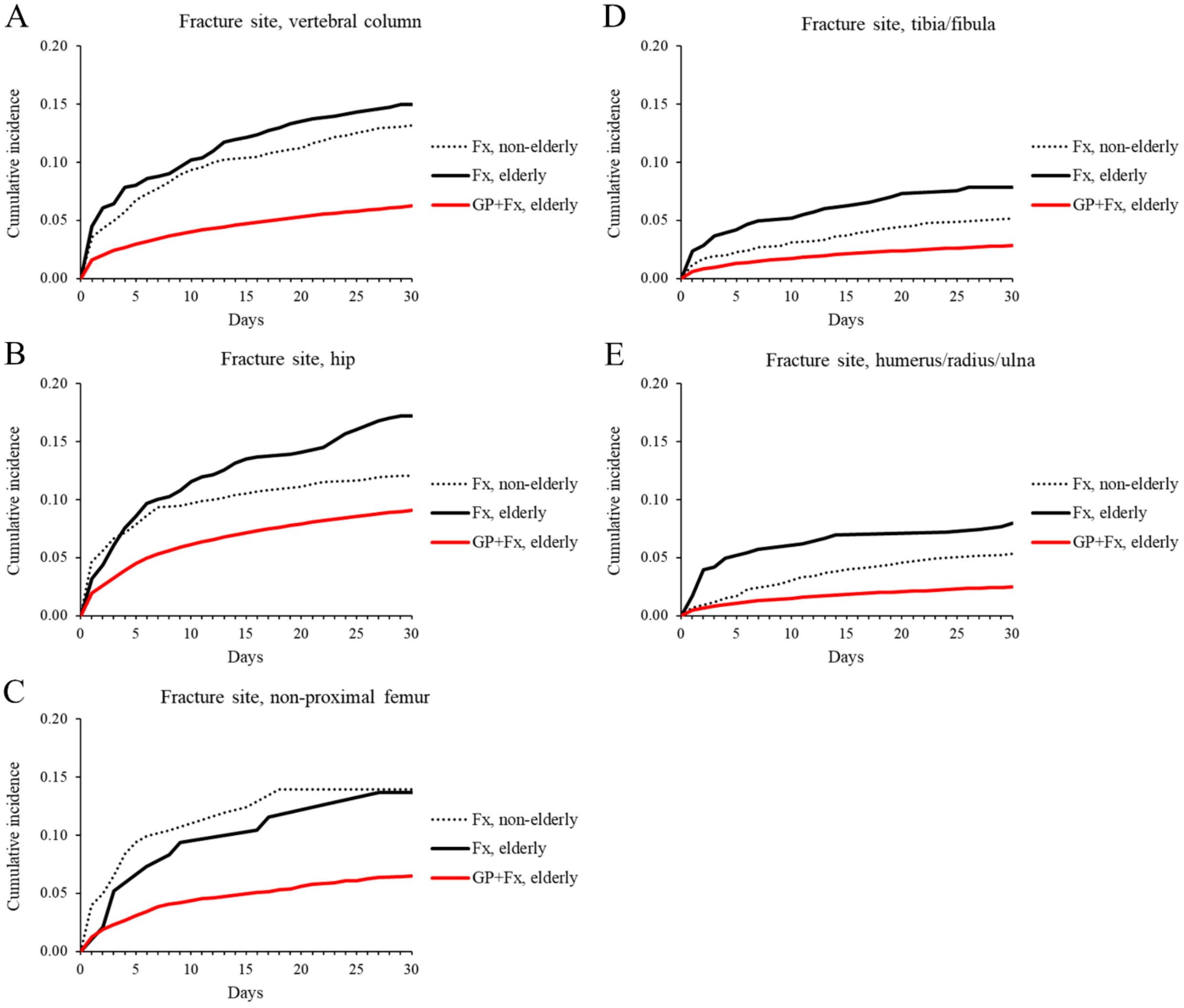

Supplementary Figure 3. Cumulative incidence of pneumonia over 30 days for (A) young, (B) middle-aged, and (3) elderly adults with intellectual disabilities that sustained a fragility fracture (Fx; n=6,183) or that did not sustain a fracture (w/oFx; n=12,366) stratified by presence of motor dysfunction.

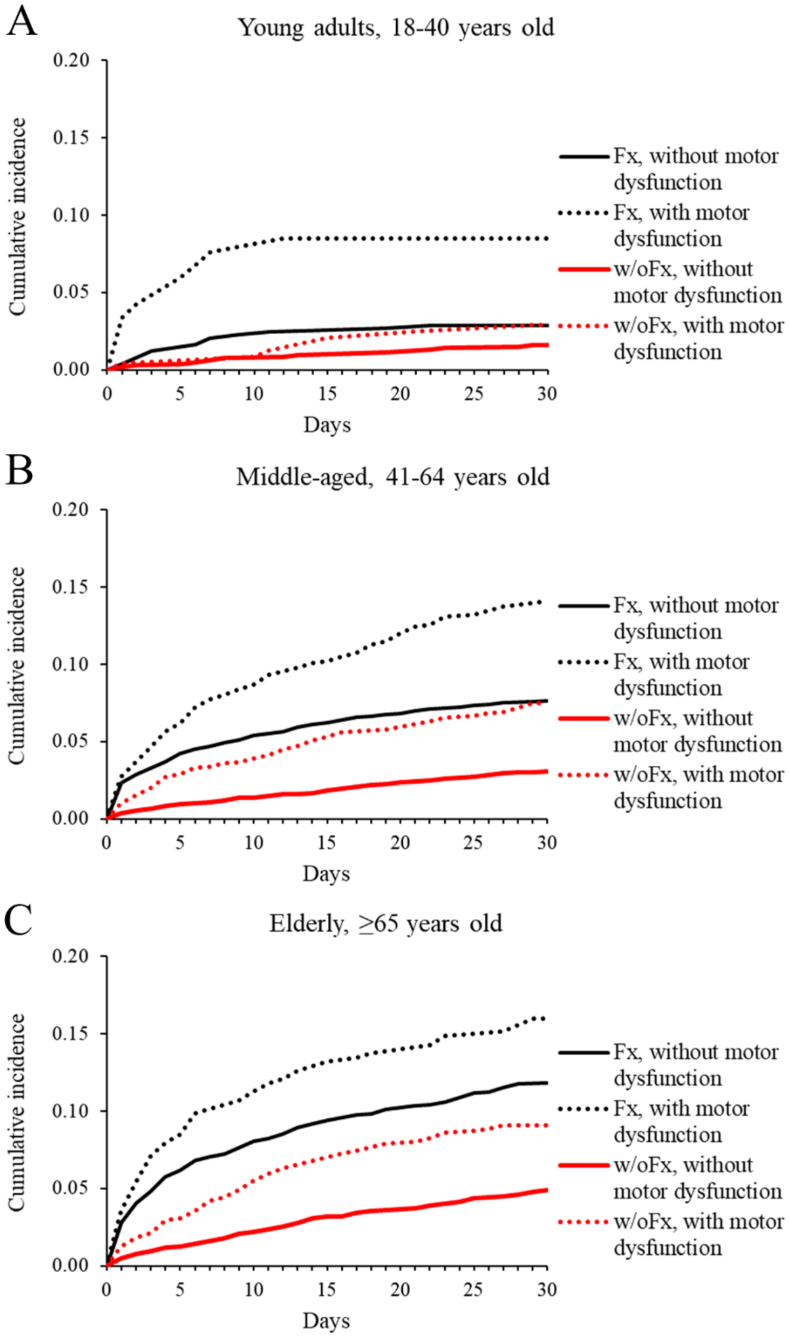

Supplementary Figure 4. Time-varying coefficient plot for 31-365 day mortality for time-varying pneumonia that violated the proportional hazards assumption.

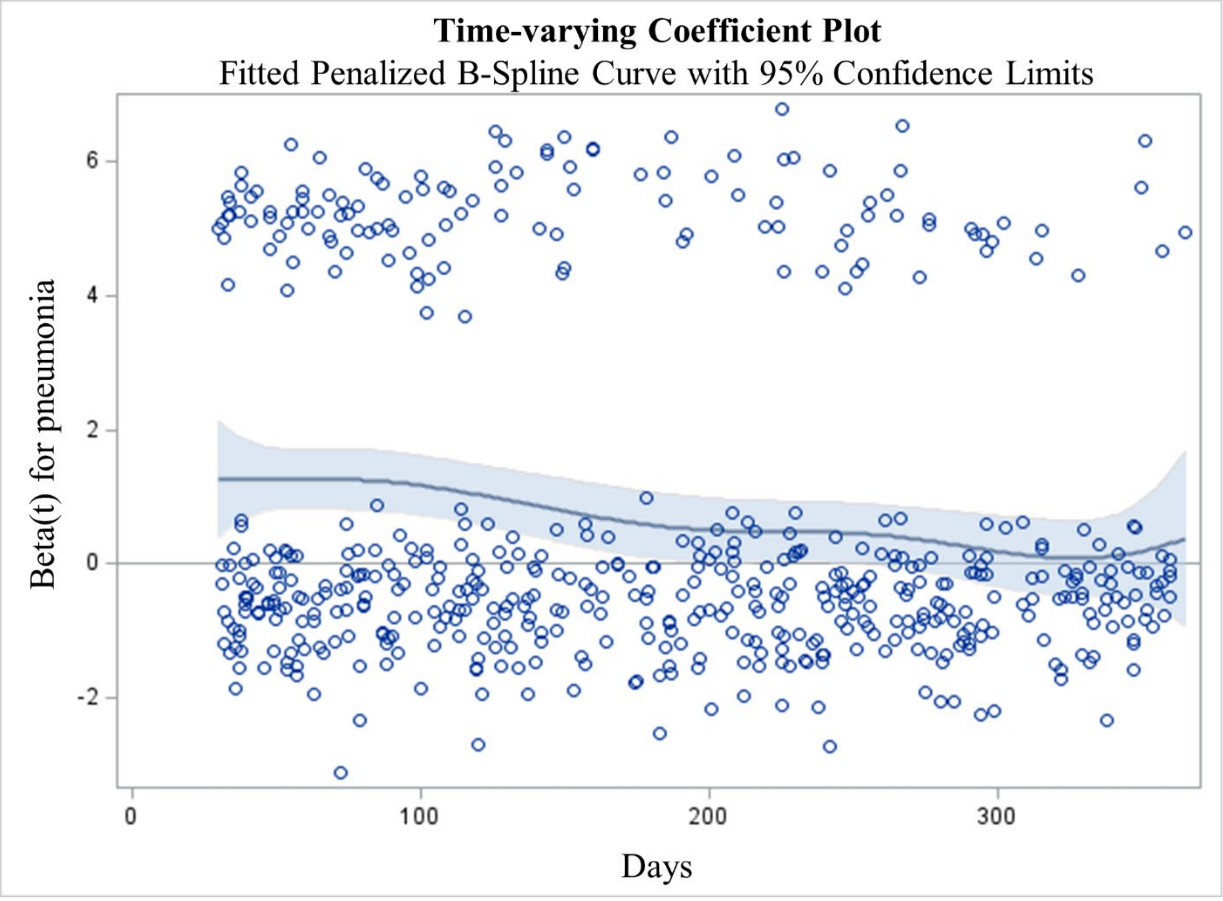

Supplement: Supplementary file 1 [file Data_Sheet_1.PDF]
